# Knowledge and attitude about organ donation and transplantation among Omani university students

**DOI:** 10.3389/fpubh.2023.1115531

**Published:** 2023-05-25

**Authors:** Nasar Alwahaibi, Anas Al Wahaibi, Mohammed Al Abri

**Affiliations:** Department of Biomedical Science, College of Medicine and Health Science, Sultan Qaboos University, Muscat, Oman

**Keywords:** attitude, knowledge, organ donation, transplantation, university students, Oman

## Abstract

**Background:**

Despite the importance of organ donation and transplantation in improving the quality of life, still, there is a shortage of organ donations, worldwide. Lack of knowledge among the public could be the reason. In previous studies, the focus was predominantly on medical students at universities. The aim of this study was to assess university students’ knowledge and attitude about organ donation and transplantation among different colleges at the university.

**Method:**

A cross-sectional study was conducted among university students between August 2021 and February 2022 using a validated self-designed questionnaire. The questionnaire consisted of five sections. The first section was about the research information. The second section was informed consent. The third section was about sociodemographic information. The fourth section was about the knowledge of organ donation. The last section was about the attitude toward organ donation. The data were analyzed by descriptive statistics and chi-square tests.

**Results:**

The study included 2,125 students. 68.1% were females, and 93.1% were in the age group 17–24 years old. Only 34.1% had good knowledge about organ donation, 70.2% had a low attitude, and 7.53% had adequate information about brain death. The most common reason for supporting donating organs among university students was to save a life (76.8%) and the most common reason for refusing organs, was I am still unaware. In addition, only 25.66% of the participants had a high attitude toward people with poor knowledge about organ donation. The majority of the students (84.13%) used online sources and social networks as the primary sources of information about organ donation.

**Conclusion:**

The knowledge and attitudes of university students toward organ donation and transplantation were low. Saving a life was the most common reason for supporting organ donation, and knowledge was the biggest obstacle. Online sources and social networks were the primary sources of knowledge. The attitude was greatly influenced by knowledge. Organizing campaigns, and events, and incorporating organ donation and transplantation into university curricula will increase university students’ knowledge and attitudes.

## Introduction

Organ donation is the contribution of a human organ or tissue from an alive or dead person to a living recipient that needs transplantation (1). The implantation of an organ is probably the only effective management for organ failure (2). Worldwide, kidney transplantation is the most commonly performed procedure, as many patients have end-stage kidney disease (ESKD). Other less common organ transplantations include the liver, heart, pancreas, lungs, and intestine. Globally, chronic kidney disease and ESKD continue to increase in both developed and developing countries (3). In general, organ transplantation improves the lifespan of patients, minimizes morbidity, improves the quality of life, permits social and medical recovery, and cuts the costs associated with medical care (4).

Despite the importance of organ donation in improving the quality of life, there has been a shortage of donated organs worldwide. This is because the number of patients on the waiting list is rapidly increasing, but the number of organ donors is not keeping up to meet the demand (5). This unbalance between the available organ donations and the patients who require organ transplantation is interrupted, which generates a severe problem in the medical field, and subsequently increases the death rate. Ethical, cultural, legal, and religious issues as well as age, gender, education, and income are reported to be the main barriers to donating organs (6–8).

A high level of knowledge and positive attitude toward organ donation and transplantation should be improved and concentrated upon in the general population to create an increase in the number of organ donors. Most university students have strong educational backgrounds, come from multiple regions, are familiar with technology, and have a lot of knowledge and experience. This makes them an important and unique group in society. In many previous studies, knowledge and attitudes regarding organ donation and transplantation were evaluated only among medical university students (9–15). Students at other colleges rarely study. Therefore, this study aimed to assess the knowledge and attitude about organ donation and transplantation among different nine colleges at the university.

## Methods

### Ethical consideration

This study was approved by the Medical Research Ethics Committee, College of Medicine and Health Sciences, Sultan Qaboos University, Oman, with an ethical approval number EC/397/2021. Preceding the study, a detailed procedure of the study was explained, and each student signed a voluntary consent form.

### Study design

This cross-sectional observational study was conducted between August 2021 and February 2022. The study was conducted in nine colleges at Sultan Qaboos University, including Medicine and Health Sciences, Engineering, Agricultural and Marine Sciences, Economics and Political Science, Science, Nursing, Law, Education, and Arts and Social Sciences. The inclusion criteria includes all local students of any age and year of study. The exclusion criteria include all non-local students and those in semester one of the foundation programs. Due to the COVID-19 pandemic, the questionnaire was conducted online *via* Google forms. The survey was distributed through emails, social media, and self-administered. The Questionnaire contains some self-developed questions, and others were obtained from literature reviews (16, 17). Additionally, the questionnaire was available in both English and Arabic versions.

### Sample size calculation

The sample size was calculated by using the formula *n* = NZ2p (1-p)/{d2 (N-1) + Z2p (1-p)}, where *n* = sample size, *N* = total number of SQU students, Z = standard normal deviate = 1.96 with a confidence level of 95%, *d* = permissible error on each side of 2% and *p* = prevalence. Because this was the first study at Sultan Qaboos University, the prevalence (*p*) was obtained from a pilot study in which the prevalence of knowledge and attitude about organ donation was 50.0%. Moreover, 5% was added to the sample size to avoid any incorrectly filled-out questionnaires. A pilot study was conducted among 15–20 students from each college who fulfilled the research criteria. Those who participated in the pilot study were excluded from the study. The Cronbach’s alpha for the reliability of the questionnaire was 0.606.

### Data collection

The questionnaire consisted of five sections. The first section was about the research information. It includes a brief description of the study, how it runs, its purpose, the importance of participation in the study, and the ethical approval. The second section was informed consent. The third section was about sociodemographic information. It contained information about sex, age, residence, college, marital status, source of information about organ transplantation, and year of study. The fourth section was about the knowledge of organ donation, and it focused on organ donation, the age limit for organ donation, the advantages, and risks of donating an organ, the possibility of donating whole or part of an organ, the presence of a national program, and organ registry. The last section was about the attitude toward organ donation, and this part concentrated on the student’s attitude toward organ donation, the role of organ donation in saving lives, registration as a donor if national registration is available, donation after death, promotion of organ donation among their relatives and the barriers for unwillingness to donate. The Questionnaire was designed in such a way that it featured a mix of positive and negative questions, allowing the study to prevent false-positive results due to incorrectly filled questions.

### Scores of knowledge and attitude

Fifteen questions were used to assess the knowledge about organ donation, and the answers were yes, no, and I do not know. The scores ranged from zero to 15. The students who scored <60% (0–8 out of 15) were considered as poor knowledge, whereas those who scored ≥60% (9–15 out of 15) were considered as good knowledge (17). The attitude was assessed by eight questions answered by yes, no, or I do not know and two multiple-choice questions. Similar scores, as with knowledge, were used to score the attitude. Achievement of 60% or more is regarded as a high attitude, and less than 60% is a low attitude. The two multiple-choice questions were used to determine what makes the participants willing or refusing to donate organs.

### Data analysis

The data were analyzed using Statistical Package for Social Science (SPSS) version 27 software (SPSS Inc., Chicago, United States). Frequencies and percentages were used to represent categorical data such as gender, age, marital status, academic degree, and college type. Continuous data were presented as mean and standard deviation. A Chi-square test was performed to measure the significant association between sociodemographic data and knowledge and attitude about organ donation. The *p*-value was considered significant if it was equal to or less than 0.05.

## Results

In total, 2,173 participated in the study. However, 48 students were excluded because they did not fulfill the research criteria. Therefore, 2,125 students were included in this study.

Only 31.9% of the students were males and 68.1% were females. Most of the students were in the age group 17–24 years old (93.1%), 94.9% were singles, and only 4.9% were married. Furthermore, only 61 (2.9%) students in the foundation program participated in the study, and more than half (52.4%) were in the foundation program’s third semester. Surprisingly, the number of master’s and Ph.D. students was low, 4.6 and 0.8%, respectively, when compared to the bachelor’s students, 94.5%. The College of Science had the highest number of participants (17.9%), followed by the College of Art and Social Sciences with 14.2%, the College of Education with 13.7%, and the College of Economics and Political Science with 12.7% (Table 1).

**Table 1:** The sociodemographic characteristics of the students toward organ donation and transplantation.

	Characteristics	Number (2125)	Percent
Gender	Males	678	31.9
	Females	1,447	68.1
Age groups (years)	17–24	1978	93.1
	25–32	92	4.3
	>32	55	2.6
Marital status	Single	2017	94.9
	Married	104	4.9
	Widow/Divorce	4	0.2
Students in the foundation program	No	2064	97.1
	Yes	61	2.9
Academic degree	Bachelor’s degree/MD	2009	94.5
	Master’s degree	98	4.6
	Ph.D.’s degree	18	0.8
Colleges	Medicine and Health Sciences	225	10.59
	Engineering	222	10.45
	Agricultural and Marine Sciences	149	7.01
	Economics and Political Science	270	12.71
	Nursing	127	5.98
	Law	159	7.48
	Science	380	17.88
	Education	291	13.69
	Arts and Social Sciences	302	14.21

MD, Doctor of Medicine. Statistical significance by Chi-square test.

About two-thirds (65.9%) of the students had poor knowledge about organ donation. Most of the students heard about organ donation (98.5%), and 95.3% of them knew that organ donation would save other people’s lives. 65.6% of the students knew that the kidney is the most transplanted organ globally. Interestingly, 82.1% of the students knew that rejection of the transplanted organ is possible, and only 14.6% knew that donor and recipient’s blood groups must be comparable and not identical. Half of the students (50.9%) knew that Islam permits organ donation. However, surprisingly, 50.1% of the students were unaware of the country’s rules regulating organ donation. Despite that, according to 73.8% of the students, organ donation is ethically acceptable.

70.8% of the students heard about brain death. However, only 15.2% of students were aware that organ donation from brain-dead patients is possible, which is equal to the percentage of students who heard about organ donation cards for brain-dead patients (15.3%). More than half of the students (55.6%) did not know that brain death is caused by stopping brain stem reflexes, and only around 12.6% of the students knew that brain death is irreversible (Table 2).

**Table 2:** University students’ responses to knowledge levels about organ donation.

		Yes	No	I do not know
1	Have you heard about organ donation?	98.5% (2093)	1.5% (32)	–
2	Would organ donation save other people’s lives?	95.3% (2026)	1.0% (21)	3.7% (78)
3	Do you know anyone who has donated an organ?	22.2% (472)	77.8% (1653)	–
4	Do you know anyone who received or is waiting to receive a kidney or other organ?	37.6% (799)	62.4% (1326)	–
5	Is the kidney the most transplanted organ in the world?	65.6% (1395)	1.4% (30)	32.9% (700)
6	Rejection of organs after transplantation is possible.	82.1% (1744)	1.9% (41)	16.0% (340)
7	The donor and recipient’s blood groups must be comparable and not identical.	14.6% (311)	43.9% (933)	41.5% (881)
8	Did the “Islamic Fatwa” allow organ donation?	50.96% (1083)	3.29% (70)	45.74% (972)
9	Are there any laws regarding organ donation and transplantation in your country?	47.86% (1017)	2.07% (44)	50.07% (1064)
10	Do you think that organ donation is ethically acceptable?	73.79% (1568)	4.66% (99)	21.55% (458)
11	Have you heard about brain death?	70.76% (1504)	29.24% (621)	–
12	Did you hear about organ donation from brain-died patients?	15.20% (323)	23.44% (498)	61.36% (1304)
13	Have you heard about organ donor cards for brain-dead patients in your country?	15.27% (324)	84.73% (1801)	–
14	Is brain death caused by the stopping of brainstem reflexes?	20.06% (426)	24.30% (516)	55.64% (1183)
15	Is brain death irreversible?	12.62% (268)	26.70% (567)	60.68% (1290)

Males were higher in poor knowledge compared to females, 71.5 and 63.2%, respectively. 72.5% of the College of Education students had poor knowledge about organ donation. Interestingly, a nearly equal percentage of students in the College of Agricultural and Marine Sciences and the College of Art and Social Sciences had poor knowledge about organ donation, 67.8, and 67.2%, respectively. On the other hand, the students in the Colleges of Nursing and Medicine and Health Sciences showed that 45.7 and 46.7%, respectively, had good knowledge about organ donation. The students in semesters 1–3 and semesters 4–6 had poor knowledge, whereas in semesters 7–9 and 10–12, students showed better knowledge. Surprisingly, the graduated students showed a high percentage of poor knowledge (67.6%), equal to the percentage of poor knowledge in semesters 1–3 and 4–6 students (Table 3).

**Table 3:** The association between sociodemographic variables and university students’ knowledge about organ donation.

Sociodemographic characteristics		Knowledge about organ donation				*P*-value
Sex		Poor		Good		
		Number	Percent	Number	Percent	
	Male	485	71.5	193	28.5	0.001
	Female	915	63.2	532	36.8	
Age groups	17–24	1,315	66.5	663	33.5	0.102
	25–32	53	57.6	39	42.4	
	>32	32	58.2	17	41.8	
College	Education	211	72.5	80	27.5	0.0001
	Economics and Political Science	191	70.7	79	29.3	
	Engineering	159	71.6	63	28.4	
	Agriculture and Marine Sciences	101	67.8	48	32.2	
	Science	249	65.5	131	34.55	
	Art and Social Sciences	203	67.2	99	32.8	
	Law	97	61.0	62	39.0	
	Medicine and Health Sciences	120	53.3	105	46.7	
	Nursing	69	54.3	58	45.7	
Academic degree	Bachelor/MD	1,328	66.1	681	33.9	0.429
	Master and Ph.D.	72	62.1	44	37.9	

MD, Doctor of Medicine. Statistical significance by Chi-square test.

University students had a poor attitude toward organ donation and transplantation, with 70.2%, while only 29.8% had a good attitude. Only 32.4% of the students would donate their kidneys or other organs after death, and 28.5% would register their names as donors after death. When we asked the students if a member of their family develops kidney failure, will they donate their kidney to him or her? 75% of the students agreed to donate. When it comes to commercial transplantation, 62.9% of the students refused to sell their organs for the sake of money (Table 4).

**Table 4:** University students’ responses to attitude levels about organ donation.

		Yes		No		I do not know	
		Percent	Number	Percent	Number	Percent	Number
1	Would you donate your kidneys or other organs after death?	689	32.4	370	17.4	1,066	50.2
2	If a member of your family develops kidney failure, will you donate your kidney to him or her?	1,594	75.0	88	4.1	443	20.8
3	If you or a member of your family develops renal failure, would you accept a kidney from a deceased person?	1,445	68.0	190	8.9	490	23.1
4	Do you agree with organ donation from non-relatives?	1,459	68.7	211	9.9	455	21.4
5	Do you agree with organ donation if you have financial interest?	344	16.1	1,337	62.9	444	20.9
6	Do you think someday you may need an organ transplant?	310	14.6	357	16.8	1,458	68.6
7	Will you register your name as a donor after death?	606	28.5	400	18.8	1,119	52.7
8	Would you advise your family to register as donors after death?	740	34.8	414	19.5	971	45.7

The students from the College of Medicine and Health Science had the highest attitude, with 38.2%. Followed by the Colleges of Engineering, Economics and Political Science, and Nursing with 32.4, 32.2, and 32.3%, respectively. 29% of the students with a bachelor’s degree or Doctor of Medicine (MD) had a high attitude, while Ph.D. students showed only 27.8% with a high attitude. When the students were between 17 and 24 years old, 29.2% had a high attitude. The percentage of high attitude increases with students between 25 and 32 years old and more than 32 years, with 37.0 and 40.0%, respectively (Table 5).

**Table 5:** The association between sociodemographic variables and the attitude of university students.

Sociodemographic characteristics		Attitude				*P*-value
		High		Low		
		Percent	Number	Percent	Number	
Sex	Male	487	71.8	191	28.2	0.273
	Female	1,004	69.4	443	30.6	
	Agricultural and Marine Sciences	105	70.5	44	29.5	
	Art and Social Sciences	218	72.2	84	27.8	
	Economics and Political Science	183	67.8	87	32.2	
	Education	224	77.0	67	23.0	
College	Engineering	150	67.6	72	32.4	0.030
	Law	115	72.3	44	27.7	
	Nursing	86	67.7	41	32.3	
	Medicine and Health Sciences	139	61.8	86	38.2	
	Science	271	71.3	109	28.7	
	Bachelor’s degree / MD	1,422	70.7	589	29.3	
Academic degree	Master’s degree	58	59.2	40	40.8	0.051
	Ph.D.’s degree	13	72.2	5	27.8	
Age	17–24	1,400	70.8	578	29.2	0.071
	25–32	58	63.0	34	37.0	
	>32	33	60.0	22	40.0	

MD, Doctor of Medicine. Statistical significance by Chi-square test.

When asked the students about the obstacles that stop them from donating organs, 58.8% said “I am still not aware,” followed by “I am afraid” with 42.6%, and only 5.4% reported that organ donation is against the Islamic religion (Table 6). The most common reason for supporting donating organs among university students was to save a life, followed by to help a fellow person, and to become a donor for someone dear to me, with 76.8, 43.9, and 28.1%, respectively (Table 7).

**Table 6:** Reasons for refusing to donate organs among university students.

	Number	Percent
I’m still not aware	1,251	58.8
I’m afraid	905	42.6
I do not trust doctors and the way that I would be treated during hospitalization as a registered donor	637	30.0
I do not believe that the transplant would be used correctly	280	13.2
Others	101	8.5
I find it irrelevant and am not really concerned about the matter	118	5.6
Against Islamic religion	114	5.4

**Table 7:** Reasons for supporting donating organs among university students.

	Number	Percent
By donating an organ, you are saving a life, which is something that agrees with my religious beliefs	1,633	76.8
I really want to help a fellow person	932	43.9
I would only become a donor for someone dear to me	597	28.1
Islamic religion allows me to do so	460	21.6
The mass media has had a positive effect on me about becoming a donor	460	21.6
I have been affected by a family member or a friend that is a donor	176	8.3
I have been sensitized by a family member or friend that needed a transplant	148	7.0
Others	101	4.8
I need the money	82	3.9

**Figure 1:**
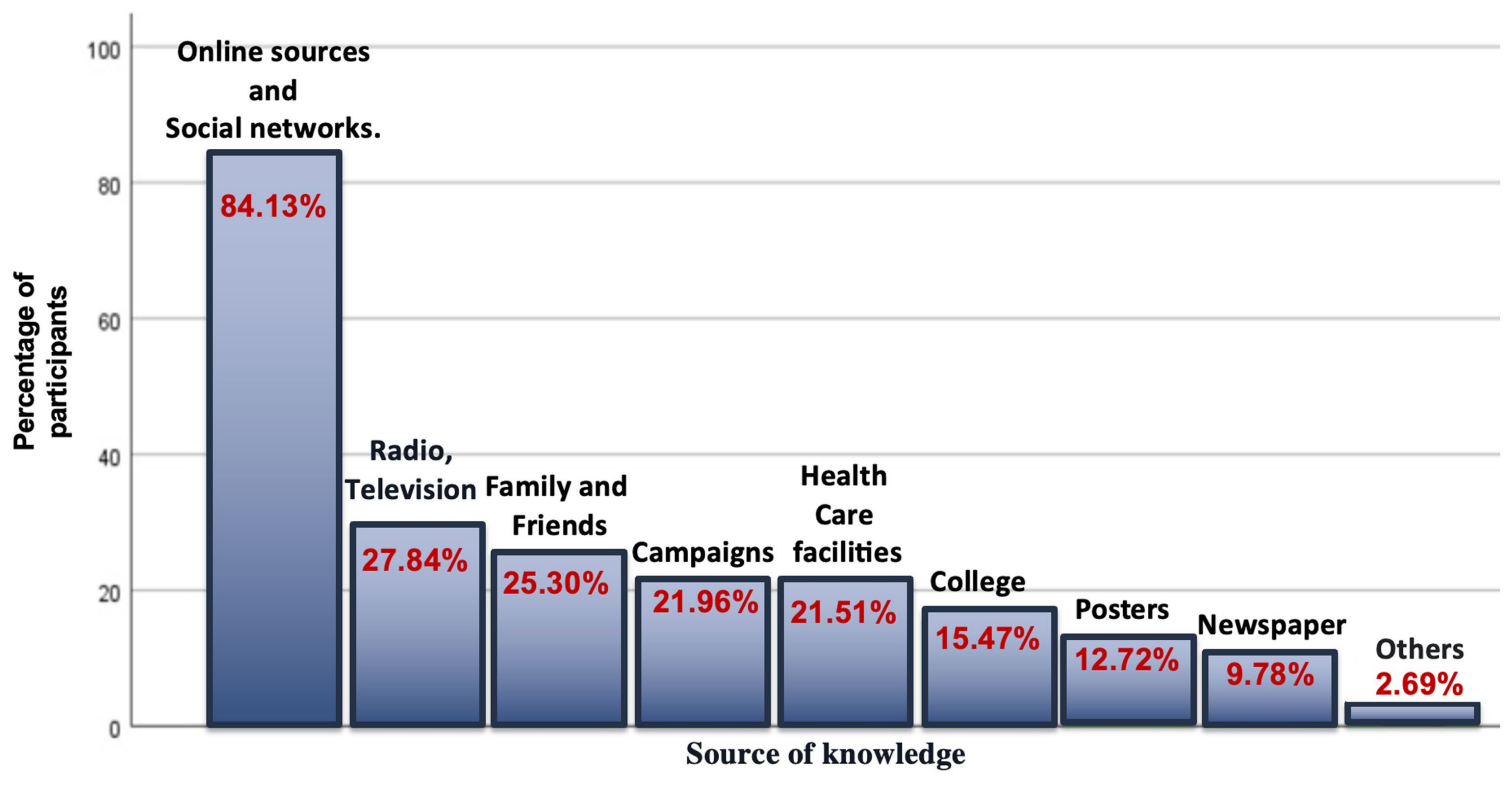
Sources of knowledge about organ donation among students.

Online sources and social networks were found to be the most common sources of information about organ donation, represented by 84.3%, followed by radio and TV 27.8%, and family and friends 25.3%, respectively. Posters and newspapers served a small role as a source of knowledge about organ donation, 12.7, and 9.8%, respectively (Figure 1).

## Discussion

University students are the strength of the country and would be able to spread the importance of organ donation among friends, families, and societies. In addition, they have more exposure to life, are more experienced, and are usually keen to help. Good knowledge and a high attitude are critical in promoting organ donation and transplantation, in particular, for patients who have no other treatment or badly suffer from other treatments. Organ transplantation supports save patients’ life and improves their survival rates (18). This study aimed to assess university students’ knowledge and attitude about organ donation.

The findings of this study showed that 98.5% of the students heard about organ donation. This finding is in line with another study conducted among medical students, which found roughly the same result, 98.5% (19). However, our study showed that only 34.1% of the students were with good knowledge about organ donation, which is lower than a study performed on the adult population in Saudi Arabia, which found that only 44.7% of the participants were with good knowledge (5). Good knowledge was shown among students from the Colleges of Medicine and Health Sciences and Nursing with 46.7 and 45.7%, respectively. Medical and nursing students are more interested in such medical issues and have more encounters with patients. This finding is in line with other similar studies (13, 20).

Furthermore, the current study found a significant association between knowledge and gender, as females (36.5%) had more knowledge than males (28.5%). Therefore, being a female is positively associated with better knowledge, which is in agreement with another study (21). However, these findings were in contrast with a study conducted on medical students in South India, where almost one-third (34.6%) of male students had adequate knowledge compared to females (24.6%) (22). In addition, our study found that the students relied heavily on online sources and social networks as a source of information about organ donation, represented by 84.13% of the total number of students and to a lesser extent on radio and television, family and friends, campaigns, health care facilities, and others. In a similar study, the internet and social media were identified as the most common sources of information about organ donation, represented by 81.2% (23). In contrast, another study showed that TV was the most important source of information about organ donation (24). Such a difference could be explained by the year in which the study was conducted. The current study was conducted in 2021/2022, and the other study was conducted in 2009. At that time, the use of the internet and social networks was minimal.

Furthermore, there was a significant association between academic semesters knowledge in which better levels of knowledge were observed with the progression in the academic semesters. However, the graduates showed lower knowledge levels compared to other students. These findings were consistent with another study (22). The current study showed that age was not significantly associated with knowledge, which is in line with other studies (21, 25).

Assessing the students’ knowledge about the Islam view on organ donation was very important as religion may affect both knowledge and attitude toward organ donation. We found that only 50.9% of the students knew that Islam allows organ donation, and it is in line with another study (16). In addition, when we asked the students about the reasons that make you refuse organ donation, only 5.4% said that it is against the Islamic religion.

The present study found that 92.5% of the students had poor knowledge about brain death. Similarly, poor knowledge was observed among 81.3% of medical students in Egypt (26). Poor knowledge about brain death consistently negatively affects organ donation (27). This suggests that students from all colleges have a low understanding of brain death. Including this information in the curriculum would increase students’ knowledge about brain death and clarify various misunderstandings. Regarding the attitude toward organ donation, this study showed only 29.8% of the participants had a high attitude. In contrast with our study, a similar study in Saudi Arabia indicated a higher attitude, with 42.4% (28). This difference can be explained by the fact that organ donation and transplantation started earlier in Saudi Arabia as well as the presence of the Saudi Center for Organ Transplantation (29). In our study, only 38.2% of the medical students had positive attitudes toward organ donation. Compared to other medical students, this finding is low. Recent study in Turkey showed that 71.2% medical students exhibited high attitudes regarding organ donation and transplantation (30). Another two studies showed that 96 and 80% of Canadian and Spanish medical students have a favorable attitude toward organ donation, respectively (31, 32).

The current study shows no relationship between sex and attitude. Thus, according to our study, being a male or female does affect your attitude. On the contrary, there was a study conducted in India showed an association between attitude toward organ donation and gender with 70.9% of the men willing to donate an organ after death while only 52.3% of the women were willing to donate an organ (33). Similar studies reported the same association (34, 35).

In the present study, the Bachelor’s degree or Doctor of Medicine (MD) students had relatively low attitudes toward organ donation (29.3%), compared with 40% of the students with Master’s degrees. However, only 27.8% of the Ph.D. students had high attitudes. That was not expected since Ph.D. students usually are more educated, but this could be explained due to very few responses from Ph.D. students.

The current study shows that age and attitude had no association. Similarly, there is no association between academic degrees and attitude. In contrast with our study, a study carried out in Poland showed an association between age and attitude. 96% of those aged less than 60 had a positive attitude toward organ donation, while 81.3% of those who were aged more than 60 years had a positive attitude (36).

This study evaluated the association between knowledge and attitude toward organ donation and transplantation. The findings show that more than 37% of those with high knowledge had a high attitude. In comparison, only 25% of those with poor knowledge had a high attitude. Thus, we can conclude that those with high knowledge had a higher attitude. This finding is in line with another study in Saudi Arabia, which showed that 40.6% of those who had good knowledge had a high attitude while only 11.1% of those who had poor knowledge had a high attitude (5).

The present study shows that the most important obstacle to organ donation was knowledge, as more than half of the participants said I am still unaware of organ donation. Moreover, the second most chosen option was, I am afraid, and that could be due to a lack of awareness. The general population, including students, may not be aware of the procedure and tests that should be taken before and after the donation. This finding is in line with another similar study, which showed that more than 60% of the adults in the general population were still not aware of organ donation (17).

Furthermore, the current study shows that most of the participants did not choose the option that says that organ donation and transplantation are against the Islamic religion. Therefore, the majority of participants are aware that organ donation and transplantation are permissible in the Islamic religion. On the contrary, a study performed in Pakistan showed that more than 45% of the participants believed that religion was the major barrier to organ donation (24).

When we asked the students what supports them to donate organs, 76.8% say that they want to save a life, and only 3.9% would do it for the sake of money. In line with this study, 70.14% of the participants in the public in China would donate organs to save lives (37). However, in Turkey, where a pilot study assessed the knowledge and attitudes of medicine, nursing, dentistry, and health technical students toward organ donation, only 35.8% would donate organs to save a life (38).

### Strengths and limitations

The strengths of our study include that students, who participated in the present study, come from different colleges, including medical, nursing, engineering, law, art, science, economics, agriculture, and education, which eliminates bias among the participants. The sample size is calculated based on a pilot study and not only estimated. However, the current study has also some limitations. First, due to the COVID-19 pandemic, we were unable to conduct face-to-face questionnaires, which would have been preferable because it would have limited inappropriately and randomly filled out questions as well as misunderstood questions, resulting in results that are more accurate. Second, this study was conducted only among one single university. Thus, to generalize the findings, the study should include students from other universities. Finally, a low number of master’s and Ph.D. students participated in this study.

## Conclusion

The knowledge and attitudes of university students toward organ donation and transplantation were low. Saving a life was the most common reason for supporting organ donation, and knowledge was the biggest obstacle. Online sources and social networks were the primary sources of knowledge. The attitude was greatly influenced by knowledge.

### Recommendations

Organizing campaigns, and events, and incorporating organ donation and transplantation into university curricula will increase university students’ knowledge and attitudes.

## Data availability statement

The original contributions presented in the study are included in the article/Supplementary material, further inquiries can be directed to the corresponding author.

## Ethics statement

The studies involving human participants were reviewed and approved by Medical Research Ethics Committee, College of Medicine and Health Sciences, Sultan Qaboos University, Oman, with an ethical approval number EC/397/2021. The patients/participants provided their written informed consent to participate in this study.

## Author contributions

NA conceived and designed the experiment, analyzed and interpreted the data, and wrote the manuscript. AA and MA performed the experiments, analyzed and interpreted the data, and wrote the manuscript. All authors contributed to the article and approved the submitted version.

## Conflict of interest

The authors declare that the research was conducted in the absence of any commercial or financial relationships that could be construed as a potential conflict of interest.

## Publisher’s note

All claims expressed in this article are solely those of the authors and do not necessarily represent those of their affiliated organizations, or those of the publisher, the editors and the reviewers. Any product that may be evaluated in this article, or claim that may be made by its manufacturer, is not guaranteed or endorsed by the publisher.
