# Supplementary material for: Knowledge and attitude about organ donation and transplantation among Omani university students
Source: Front Public Health. 2023 May 25;11:1115531. doi: 10.3389/fpubh.2023.1115531 (PMC10248022; doi:10.3389/fpubh.2023.1115531)
Supplement: Supplementary file 7 [file Data_Sheet_1.PDF]

Consent form for Research Project

Knowledge and attitude toward organ donation among SQU students

Please tick the appropriate box:

- **I have read and understood the project information sheet dated …../……/…..**
- **I have been given the opportunity to ask questions about the project.**
- **I agree to take part in the project. Taking part in the project will include completing a survey/being interviewed.**
- **I understand that my taking part is voluntary; I can withdraw from the study at any time and I will not be asked any questions about why I no longer want to take part.**
- **I understand that my words may be quoted in publications, reports, web pages, and other research outputs but my name will not be used unless I requested.**
- **I understand that other researchers will have access to this data only and they should agree to preserve the confidentiality of that data and terms that I have specified in this form.**

Name of the participant	Signature	Date

Researcher	Signature	Date
