# Supplementary material for: Knowledge and attitude about organ donation and transplantation among Omani university students
Source: Front Public Health. 2023 May 25;11:1115531. doi: 10.3389/fpubh.2023.1115531 (PMC10248022; doi:10.3389/fpubh.2023.1115531)
Supplement: Supplementary file 8 [file Data_Sheet_2.PDF]

Information Sheet for Research Project

Knowledge and attitude toward organ donation among SQU students

You are invited to take part in a research study. Before you decide whether or not to take part, it is important for you to understand why the research is being done and what it will involve. Please take time to read the following information carefully.

What is the purpose of the study?

A group of Medical students is carrying out a survey to assess the knowledge and attitude toward organ donation among SQU students. Moreover, they are trying to correlate the socio-demographic features of participants with their level of knowledge and attitude. The expected results that young people towards organ donation may have a significant impact on their preparedness to donate.

Why have I been invited to take part?

We are asking SQU students in all nine colleges to complete this survey.

Do I have to take part?

It is up to you to decide whether to take part, taking part is voluntary. If you do decide to take part, you will be given this information sheet to keep and be asked to sign a consent form. If you decide to take part, you are still free to withdraw at any time and without giving a reason.

What would I have to do?

If you decide to take part, the survey will take approximately 8 minutes to complete.

Confidentiality

All the information that is collected will be anonymous and kept strictly confidential.
All details that can identify you will be removed before storing the data.

Thank you for taking the time to read this information sheet.
